# Advances in Multidisciplinary Management of Skull Base Meningiomas

**DOI:** 10.3390/cancers13112664

**Published:** 2021-05-28

**Authors:** Tamara Ius, Alessandro Tel, Giuseppe Minniti, Teresa Somma, Domenico Solari, Michele Longhi, Pasquale De Bonis, Alba Scerrati, Mario Caccese, Valeria Barresi, Alba Fiorentino, Leonardo Gorgoglione, Giuseppe Lombardi, Massimo Robiony

**Affiliations:** 1 Neurosurgery Unit, Department of Neurosciences, Santa Maria della Misericordia University Hospital, 33100 Udine, Italy; 2 Maxillofacial Surgery Department, Department of Medicine, Santa Maria della Misericordia University Hospital, 33100 Udine, Italy; alessandro.tel@icloud.com (A.T.); massimo.robiony@uniud.it (M.R.); 3 Department of Medicine, Surgery and Neurosciences, University of Siena, Policlinico Le Scotte, 53100 Siena, Italy; giuseppe.minniti@unisi.it; 4 IRCCS Neuromed, 86077 Pozzilli, Italy; 5 Division of Neurosurgery, Department of Neurosciences, Reproductive and Odontostomatological Sciences, Università degli Studi di Napoli Federico II, 80125 Naples, Italy; teresa.somma@unina.it (T.S.); domenico.solari@unina.it (D.S.); 6 Unit of Radiosurgery and Stereotactic Neurosurgery, Department of Neurosciences, Azienda Ospedaliera Universitaria Integrata (AOUI), 37128 Verona, Italy; michele.longhi@aovr.veneto.it; 7 Department of Neurosurgery, Sant’ Anna University Hospital, 44124 Ferrara, Italy; dbnpql@unife.it (P.D.B.); scrlba@unife.it (A.S.); 8 Department of Morphology, Surgery and Experimental Medicine, University of Ferrara, 44124 Ferrara, Italy; 9 Department of Oncology, Oncology 1, Veneto Institute of Oncology IOV-IRCCS, 35128 Padua, Italy; mario.caccese@iov.veneto.it (M.C.); giuseppe.lombardi@iov.veneto.it (G.L.); 10 Department of Diagnostics and Public Health, University of Verona, 37134 Verona, Italy; valeria.barresi@univr.it; 11 Radiation Oncology Department, Advance Radiation Therapy, General Regional Hospital F. Miulli, 70021 Acquaviva delle Fonti, Italy; A.fiorentino@miulli.it; 12 Department of Neurosurgery, Hospital “Casa Sollievo della Sofferenza”, 71013 San Giovanni Rotondo, Italy; l.gorgoglione@operapadrepio.it

**Keywords:** skull base meningioma, surgery, radiotherapy, radiosurgery, systemic treatment, 3D virtual planning

## Abstract

**Simple Summary:**

The most frequent intracranial neoplasm is meningioma. About 30% of these are represented by skull base meningiomas (SBMs). Patients with SBMs can be treated with a multimodal approach based on surgery, medical treatment and radiation-based therapy; however, the gold standard treatment for the majority of symptomatic meningiomas is still surgery. Surgical intervention is performed with the goal of maximum safe resection. This, however, poses technical challenges because of the proximity of these tumors with deep critical neurovascular structures, tumoral texture and consistency. A multimodal treatment, in combination with stereotactic radiosurgery and radiation therapy, is thus of utmost importance to achieve a satisfactory functional outcome and tumor control. The aim of this review was based on the identification of optimal multidisciplinary management of patients with SBMs. The investigation includes the relevant biological and clinical characteristics, the novel therapeutic approaches, highlighting the importance of a specialized multidisciplinary team, which is mandatory for SBM management.

**Abstract:**

The surgical management of Skull Base Meningiomas (SBMs) has radically changed over the last two decades. Extensive surgery for patients with SBMs represents the mainstream treatment; however, it is often challenging due to narrow surgical corridors and proximity to critical neurovascular structures. Novel surgical technologies, including three-dimensional (3D) preoperative imaging, neuromonitoring, and surgical instruments, have gradually facilitated the surgical resectability of SBMs, reducing postoperative morbidity. Total removal is not always feasible considering a risky tumor location and invasion of surrounding structures and brain parenchyma. In recent years, the use of primary or adjuvant stereotactic radiosurgery (SRS) has progressively increased due to its safety and efficacy in the control of grade I and II meningiomas, especially for small to moderate size lesions. Patients with WHO grade SBMs receiving subtotal surgery can be monitored over time with surveillance imaging. Postoperative management remains highly controversial for grade II meningiomas, and depends on the presence of residual disease, with optional upfront adjuvant radiation therapy or close surveillance imaging in cases with total resection. Adjuvant radiation is strongly recommended in patients with grade III tumors. Although the currently available chemotherapy or targeted therapies available have a low efficacy, the molecular profiling of SBMs has shown genetic alterations that could be potentially targeted with novel tailored treatments. This multidisciplinary review provides an update on the advances in surgical technology, postoperative management and molecular profile of SBMs.

## 1. Introduction

Meningioma account for 16–36% of all intracranial tumors in adults [1]. According to the World Health Organization, these lesions are currently classified into fifteen histotypes and three grades of malignancy, of which 90% are of Grade I [2]. The most significant prognostic factors for these tumors include the histological grade according to the World Health Organization (WHO) criteria [2] and the extent of surgical resection according to the Simpson scale [3]. About 30% of intracranial meningiomas are represented by skull base meningiomas (SBMs) [4,5,6,7]. The surgical goal of radical resection is frequently hindered by the proximity of SBMs with deep critical neurovascular structures, complex vascularity, tumoral texture and consistency. In the past, the skull base was considered an inaccessible surgical location. Recent advances including the introduction of microsurgical techniques, improvements in imaging, virtual surgical simulation, and technological refinement of surgical instruments, along with the widespread use of minimally invasive approaches have radically changed SBM surgical management.

The goal of SBM surgery is the complete resection of the tumor, surrounding dura and infiltrated bone (if present), traditionally recognized as Simpson grade I resection [8]. Despite recent advances in microsurgical techniques and treatment strategies, this goal is often challenging to achieve, mainly because of the involvement of neurovascular structures and/or limited instrument maneuverability along narrow surgical corridors [9,10]. Bone infiltration or venous sinuses involvement can further limit the radical resection rate. Multimodal treatment, in combination with stereotactic radiosurgery (SRS) and/or fractionated radiation therapy (fSRT), is thus increasingly considered to achieve a satisfactory functional outcome and long-term tumor control.

Numerous studies in the literature have shown the efficiency and safety of radiosurgery in addition to the role it has as primary or adjuvant therapy [11,12,13]. The definition of molecular features, based on genetic mutations and on the methylation profile, is currently providing new insights into prognosis and treatments options [14].

The purpose of this review was to provide a general overview on SBM treatments, highlighting the role of interdisciplinary management. This paper also investigated the recent innovations in terms of preoperative virtual planning, intraoperative navigation, and postoperative adjuvant strategies.

## 2. Materials and Methods

The authors conducted a literature search in MEDLINE PubMed evaluating patients with SBM. The search considered both prospective and retrospective studies. For the most comprehensive detection of papers, the search query was built as follows using a combination of medical subject headings (MeSH): “skull base” [MeSH] AND “meningioma”[MeSH] and free text terms: “surgery” OR “surgical approach*” OR “surgical planning” OR “radiotherapy” OR “radiosurgery” OR “systemic treatment” therapy” OR “hypofractionated radiotherapy” or “chemotherapy”.

We included relevant studies published from 1970 to 2020. Only studies published in English language and including human subjects were considered. A total of 166 potentially relevant studies were identified, including 129 retrospective studies and 37 reviews.

## 3. Modern Surgical Planning

### 3.1. The Role of Computer-Aided Approaches

SBM surgery is demanding due to both the size and involvement of deep neurovascular structures such as perforating arteries, veins and cranial nerves, which are often encased or displaced by the tumor. Considering the above, a detailed surgical strategy is crucial for obtaining a maximum-allowed resection with minimal risks of permanent morbidity. The so-called “4D rule” (de-vascularization, detachment, de-bulking, dissection) is essential in SBM surgery.

Nowadays, virtual surgical planning gives the opportunity to perform highly accurate surgical approach rehearsal, greatly enhancing the preoperative workflow. Virtual planning starts with appropriate image acquisition, often requiring multiple modalities [15]: Tel et al. [16] described a multimodal image fusion algorithm based on automatic registration of CT and MRI images. For computer-reconstruction of intracranial vessels, MRI angiography is essential to analyze the three-dimensional spatial relationship between the tumor and the vessels [17,18]. Segmentation, defined as the process of automatic or semi-automatic detection of boundaries for regions of interest within DICOM images, allows anatomical and pathological structures to be identified, which are rebuilt in the three-dimensional space across all slices of the radiological image [19]. Segmentation can be performed using a combination of semi-automatic algorithms, including thresholding and region growing, and manual refinements, yielding tessellated geometrical representations named “mesh”. Geometrical models can undergo CAD (computer-assisted design) operations, including the simulation of osteotomies, removal of bone segments, tumor excision, showing a virtual representation of planned surgical maneuvers in relation to critical anatomical structures [16]. Three-dimensional geometry files, named STL (Standard Tessellation Language), can be imported in navigation systems to allow navigation of the entire virtual plan and not just raw DICOM images.

**Figure 1:**
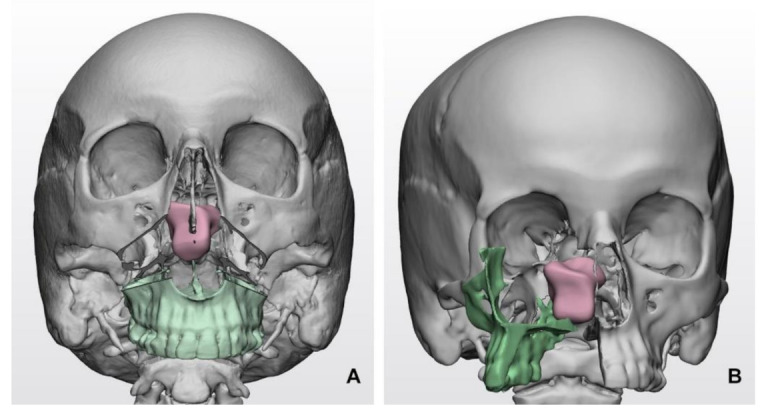
Simulated transfacial accesses using virtual models in clival meningiomas: (**A**) Le Fort I transmaxillary access; (**B**) transfacial maxillary-split approach. The bone flap is represented in green color, while the tumor mass is represented in pink color.

Virtual surgical planning plays a prominent role in simulating surgical accesses. Combined maxillofacial and neurosurgical procedures may be selected for huge SBM developing within the clival region or anterior skull base with ethmoido-orbital invasion [20]. In this setting, virtual surgical planning allows the shape and trajectory to be defined for osteotomies and the simulation of facial skeleton dismantling, paving the way for skull base fossae in a relatively compact space, dense in essential anatomical structures (Figure 1).

Virtual planning plays a crucial role in the reconstructive phases as well, as it provides a foreseeable geometrical configuration of the final anatomical geometries, allowing devices to be personalized, such as reconstructing plates and prostheses.

### 3.2. The Role of Tractography

Diffusion MRI and tractography currently represent the only way to reconstruct white matter in humans in vivo, providing a non-invasive and feasible method for evaluating the subcortical pathways changes, especially in glioma surgery [21,22,23]. In recent years, its use has been gradually spread for preoperative cranial nerve reconstruction in SBM surgery [24,25,26]. In the latter clinical setting, probabilistic tractography currently appears to be an emerging and promising tool to predict the position of displaced cranial nerves around skull base lesions [24,25].

Applying tractography to cranial nerves demands, however, advanced anatomical, radiological, and computational skills to achieve correct fiber tracking and to avoid spurious tracts. In addition, the main challenging limitations in cranial nerves tacking are represented by their small size, intricate anatomical environment sensitive to susceptibility artifacts, and a limited MRI spatial and angular resolution. Nevertheless, recent studies have demonstrated effective tracking for large cranial nerves such as optic nerve, trigeminal nerve, or acoustic-facial bundle, highlighting the potential role of tractography both in a surgical setting and intraoperative strategy [25,26].

In order to validate tractography’s effectiveness in SBM surgery, further prospective investigations are required with the aim of assessing the tracking reproducibility and the impact on patients in terms of operative time, clinical follow-up and quality of life.

## 4. The Role of Surgery

Surgical access to SBMs is one of the most challenging procedures due to the narrow surgical corridors and the proximity of these tumors to critical neurovascular structures.

Approach selection is a key-point in SBM surgery in order to manage the lesion without harming the neurovascular surrounding structures and, in the last 20 years, many different approaches have been described. Table 1 shows the main surgical approaches subclassified in three main categories (anterior, middle and posterior fossa meningioma) according to meningioma locations.

The trans-sphenoidal and the other extended transnasal approaches have revolutionized the management of meningiomas involving areas of the median and paramedian skull base surrounding the sella and cavernous sinuses regions. Selected SBMs, originating from the tuberculum sellae, planum sphenoidale, and olfactory groove, have become amenable to transnasal resection [27,28,29]. The undaunting amelioration of these novel techniques has led to these indications expanding to a wide range of SBMs. At present, “the edge of the envelope” has still not been defined [30] and the surgical possibilities of removing meningiomas of the skull base are widening [31]. In this regard, in selected cases, cooperation with maxillofacial surgeons can be useful to create adjunctive surgical corridors through facial incisions or bone osteotomies [32,33].

In selected cases, the cooperation between neurosurgeons and maxillofacial surgeons in SBM surgery might be twofold, representing an aid to create wider surgical accesses as well as to perform a more radical resection. This latter point should represent the choice for patients presenting with bulky tumors invading the nasal cavity or intraorbital space.

Therefore, bulky disease with infiltration of orbits and ethmoid requires extensive resection for which wider surgical exposure is achieved through transfacial or transoral approaches. In detail, such approaches allow for a wide exposure of the ethmoidal cells and orbital compartment, enabling resection of masses extending downward beyond the cribriform plate or invading the orbit [34,35].

Middle and posterior cranial fossa approaches represent a very flourishing field that have been proposed and intermittently preferred over the years. The approaches so far described go from the standard subtemporal or retrosigmoid approaches [36,37,38,39,40] to more complex and extended ones, and/or also combined with wider skull base bone removal [41,42,43]. Indeed, bony structure removal does not always correlate with a better surgical maneuverability or a reduced parenchymal retraction [44,45]. The best approach needs to be tailored to each patient based upon several peculiar factors (pathological, anatomical, functional and reconstructive). Several studies have demonstrated a strong correlation between the extent of resection and the frequency of recurrence in SBMs, highlighting the central role of surgery in their workflow [11,46,47,48].

There is no general consensus in considering SBMs genetically different from their non-SBM counterparts [8]; continuous refining of operative techniques is thus required to obtain satisfactory long term outcomes Complete surgical resection is the goal of SBM surgery, but can seldom be limited because of the intimate relationships with the brain stem, neurovascular structures and cranial nerves [49,50]. Therefore, the surgical aggressiveness needs to be weighed against risks of morbidity, tumor biology, patients age, functional status and functional expectations [13,51].

**Figure 2:**
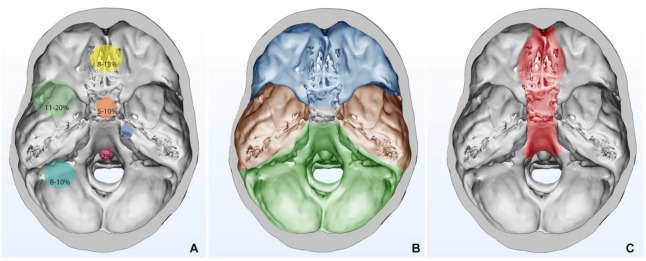
(**A**) Relative frequencies in SBM locations are stratified and shown with circles of progressively wider diameter; (**B**) Topological subdivision of skull base: anterior skull base is shown in blue, middle skull base is shown in brown, and posterior skull base is shown in green; (**C**) Anatomical area for which endoscopic endonasal approach can be used.

The approaches can be extended and combined in relation to the meningioma’s size and surgeon’s choice (Table 1, Figure 2). Appropriate knowledge of surgical anatomy, adequate corridors, release of CSF, comfortable and precise microsurgical instrumentation are the key concepts in SBM surgery, resulting essential for the patient outcomes.

### 4.1. Intraoperative Neurophysiological Monitoring in Skull Base Meningiomas

Nowadays, there is an increasing interest in the role of intraoperative neurophysiological monitoring (IONM) in SBM surgery.

The surgical resection of large meningioma, especially when encasing nerves and/or the main cerebral vascular trunks and/or their perforating vessels skull base, requires extensive maneuvers that can lead to pyramidal tract impairments or cranial nerves palsy [52,53]. Advances in anatomy, microsurgery, neuroimaging, and intraoperative monitoring have gradually reduced the incidence of cranial nerve palsy [53,54]. The IONM strategy during SBM surgery has to be tailored according to tumor location and the vascular and neural structures involved. IONM details are reported in Table 1 [4].

### 4.2. Preoperative Embolization

Devascularization of the lesion remains an important goal in meningioma surgery, to be achieved before other maneuvers. Differently from meningiomas of the convexity, those arising at the skull base have deep, poorly accessible feeding vessels. Preoperative embolization might ease surgical dissection, inducing a lower risk of intraoperative blood loss, and as a consequence, decreasing the surgical morbidity. Preoperative embolization of SBM is still, however, a controversial and debated issue, also because of the poor effectiveness and the risk of complications related to inadvertent occlusion of off-target arteries [55,56,57].

The preoperative embolization indication depends mainly on the meningioma’s size and location. The most commonly cited indications for pre-operative embolization include size >4 cm, high vascularity, and the convexity site for meningioma being supplied primarily by the external carotid artery. However, in selected cases, embolization may also result as useful for SBM meningiomas in which the arterial supply is deep and not reached until the late phases of tumor debulking [58].

As a general rule, amongst factors that deter preoperative embolization, ease of vascular access intraoperatively, dangerous external carotid artery–internal carotid artery anastomosis, the presence of feeders to cranial nerves, internal carotid artery predominant blood supply (>50% on angiography), and high tortuosity or narrowness of the feeding vessels are included [59]. Until further evidence from clinical trials emerges, the decision to preoperatively embolize a meningioma should be tailored for each patient according to tumor size, location, and estimation of degree of blood loss [58,59].

### 4.3. Reconstruction of the Surgical Route

Reconstruction of the anterior skull base has the main role of restoring the separation between the intracranial and the extracranial space, in particular to prevent leakage of CSF and related threatened complications, above all meningitis. A variety of techniques have been described [60], accounting for the use of dural substitutes and local flaps, used to restore separation between the brain and extracranial space [61]. In the case of wider defects, free flaps also represent an option [62].

Amongst local flaps, it is worth mentioning the nasoseptal flap, described by Hadad et al., because of its impact on endoscopic surgery, as it provides a minimally invasive and effective method to repair anterior skull base defects [63]. It consists of a vascularized mucoperichondrial/periosteal flap harvested from the nasal septum, which is pedicled on the posterior septal branch of the sphenopalatine artery and can be mobilized and transposed on the defect using an entirely endoscopic approach.

For transfacial accesses requiring the disassembly of facial subunits, reconstruction follows the same principles of fracture treatment using internal rigid fixation with titanium miniplates and miniscrews. Recently, supporting the experience of maxillofacial surgeons, the use of CAD-CAM technology has been introduced in clinical practice to simulate the reconstruction of skull base defects.

Moreover, the widespread distribution of virtual planning software in laboratories embedded in modern hospitals makes such processes more affordable and contributes to shared knowledge on technology. Nowadays, models for pre-operative planning [64] or reconstruction of parts of the skull base or the facial skeleton can be performed “in-house”, using commercially available technology, by 3D printing of molds for PMMA modelling according to the desired plan [65]. Nevertheless, reconstruction of the skull base using a prosthetic device, although customized, is generally difficult, and few examples are documented. If the lesion extends through the lateral skull base into the glenoid fossa of the temporomandibular joint (TMJ) causing joint dysfunction, concomitant skull base and TMJ replacement has been described using a customized TMJ prostheses extended to the lateral skull base [66]. As for the anterior fossa, reconstruction across the cribriform plate is usually performed using a soft tissue flap, whereas the orbital roof can be reconstructed using customized alloplastic implants, which offer the maximum accuracy in replicating the original anatomy. Alternatively, a titanium mesh can be prebent over a 3D printed template to provide a customized implant at a considerably lower cost [67,68].

## 5. Histopathological Features

Several studies have demonstrated that meningiomas at different anatomical sites have diverse histological and genetic features [69,70] (Table 2), which could provide relevant prognostic information and open the perspective to novel target therapies.

SBMs mainly show the meningothelial histotype, and compared to non-skull based ones, they have a lower incidence of grade II/III histology (8.6–20% vs. 40%) and of *NF2* alterations (20% vs. 46%), and a higher incidence of secretory histotype (63% vs. 37%) [60,62,63], a rare grade I variant, characterized by peritumoral edema [71]. Then, SBMs can be further categorized, as those localized at the lateral and posterior skull base mainly feature *NF2* impairment [72,73], while those at the anterior and middle skull base are *NF2* wild type and may have mutations in other genes, including *AKT1*, *PIK3CA, SMO, TRAF7, KLF4* and POLR2A [69,72,73,74]. In detail, around 15% of skull base meningiomas have alterations in the *PI3K/AKT/mTOR* signaling pathway, consisting of *AKT1^E17K^* and *PIK3CA* mutations, in association with meningothelial histotype or brain invasion [69,74,75]. About 28% of meningioma at the middle anterior skull base, and, specifically, at the olfactory groove, have an impaired hedgehog pathway due to *SMO* mutations (L412F and W535L), which are mutually exclusive to *AKT1* mutations [69,72,73,75,76,77]. These latter tumors mainly have meningothelial histotype and a low mitotic index, but a significantly higher recurrence rate than *AKT1*-mutated meningiomas at the same site [77]. A proportion of meningiomas at the middle skull base (ranging between 2.1% and 24%, and between 8.6% and 11.8%, respectively) were reported to display *TRAF7* and *KLF4*^K409Q^ mutations [69,75,76], which co-occur in secretory meningiomas, and which may coexist with *AKT1* mutations [69,73,75,76]. Finally, there is a distinctive group of skull base meningiomas, originating at the tuberculum sellae and with meningothelial histotype, that are characterized by mutations of *POLR2A*, which encodes for the catalytic subunit of Polymerase RNA II (DNA directed) polypeptide A [78].

## 6. Radiation Therapy

### 6.1. Fractionated Radiotherapy

Postoperative radiation therapy (RT) using doses of 50–55 Gy in 30–33 fractions has been frequently used for benign SBMs, either after incomplete resection or tumor progression. Local control rates from 75 to 90% at 10 years have been reported following conventional RT and 3D conformal RT (Table 3) [79,80,81,82,83,84], equivalent to that observed after complete resection, and better than that achieved with subtotal resection alone [85]. Similar tumor control has been observed for patients receiving postoperative RT or at the time of tumor recurrence/regrowth [81,82,83].

The reported treatment-related toxicity is relatively low and includes the development of neurological and endocrinological adverse events (Table 3). Radiation-induced optic neuropathy, presenting as decreased visual acuity or visual field defects, occurs in less than 5% of irradiated patients with an SBM. Deficits of cranial nerves passing through the cavernous sinus, which include the oculomotor nerve, trochlear nerve, abducens nerve, and the V1 to V2 branches of the trigeminal nerve have been reported in 1–4% of patients when radiation doses do not exceed 54 Gy in conventional fractionation 1.8–2.0 Gy daily [79,80,81,82,83,84]. Similarly, the risk of radionecrosis remains exceptional for doses less than 60 Gy. Hypopituitarism is reported in up to 20% of patients, with higher risk for large SBMs invading the pituitary sella. Neurocognitive dysfunction has been occasionally reported in irradiated patients with large meningiomas, especially impairment of short-term memory [80,86,87,88].

Postoperative fractionated RT using doses of 59.4 Gy in 13 fractions of 1.8 Gy per fraction is typically recommended after surgical resection of Grade II and Grade III meningiomas [89,90]. Cooperative group studies RTOG 0539 and EORTC 22042 support the role of early postoperative RT in patients with WHO grade II meningiomas after subtotal resection and grade III meningiomas with any resection extent. However, the benefit of RT in terms of survival and local tumor control following complete surgical resection remains a matter of debate. The recently closed ROAM/EORTC randomized trial will clarify the role of adjuvant radiotherapy in reducing the risk of tumor recurrence following complete surgical resection of atypical meningioma [91].

Over the last few decades, RT has seen technological advances through all the steps involved in radiation treatment with improvement in the accuracy of target delineation, treatment planning process and delivery [92]. Modern radiation techniques, including fractionated stereotactic radiotherapy (fSRT), intensity-modulated radiotherapy (IMRT) and volumetric modulated arc therapy (VMAT), allow for more precise treatments as compared with conformal RT, while reducing radiation exposure to surrounding sensitive brain structures. Table 3 shows a summary selected series using either fSRT or IMRT [79,80,81,82,83,84,93,94,95,96,97,98,99,100,101,102,103]. With a median follow-up of 42–107 months, the reported actuarial median local control ranges from 93 to 100% at 5 years and 91.5 to 100% at 10 years.

A clinical neurological improvement is reported in 14–44% of patients after fSRT [94,95,96,97,98,99,100], with acceptable late significant toxicity. With doses of 50–55 Gy in 1.8.2.0 Gy per fraction, pituitary hormone deficits occur in less than 15% of patients. The development of optic neuropathy or other cranial deficits is reported in less than 3–4% of patients. For patients treated with conventionally fractionated RT, the analysis of prognostic factors showed that tumor size was a predictor of tumor control [79,80,95,97,100,104]. In 54 patients with SBMs who received conventional RT, Connell et al. [104] observed 5-year tumor control rates of 93% for lesions more than 5 cm and 40% for lesions less than 5 cm; similar results have been reported by others [79,80,95,97]. In some, but not all, studies, clinical outcome was similar for patients treated with early postoperative RT or at the time of tumor progression. With regard to the radiation dose, no outcome differences have been reported following doses of 50–54 Gy or >54 Gy.

In addition, few studies have compared the outcome of SRS and fSRT in SBMs [97,105,106,107]. In a large retrospective study of 927 patients from three German centers treated with either SRS (median dose, 13 Gy) or fSRT (median dose, 54 Gy/30 fractions) for meningiomas, Combs et al. [97] reported local control rates of 92% at 5 years and 86% at 10 years, with no difference between techniques. Among patients treated with fSRT, there was no difference between 54 Gy and 57.6 Gy. Side effects were below 5% after either SRS or fSRT, without any severe treatment-related complications. In another series of 51 treated with fSRT and 77 who had SRS for a SBMs, Torres et al. [105] showed tumor control rates of 97% for patients with a median follow-up of 24 months and 90% for those with a median follow-up of 40 months. Late toxicity was observed in 5% of patients treated with SRS and 5.2% patients who received fSRT.

Some retrospective studies have reported the use hypofractionated SRT for SBMs, as shown in Table 4 [98,100,108,109,110,111]. Using doses of 21–25 Gy delivered in 3–5 fractions, the observed local control in six studies including 337 patients is 93–95% at 5 years, with a reported cranial nerve toxicity of less than 5%. In a large retrospective series of 168 patients receiving CyberKnife-based hypofractionated SRT for SBMs, Marchetti et al. [111] showed a local control rate of 95% at 5 years with a toxicity rate of 3.7%, and similar results have been observed in a few other studies using either CyberKnife or LINAC technologies. In a systematic review on the clinical outcomes of hypofractionated SRT for intracranial meningiomas including 630 patients reported in fourteen studies published between 2004 and 2016, Nguyen et al. [112] reported a crude control of 90–100% with median late toxicity rates of about 10%.

Based on several retrospective studies, hypofractionated SRT may represent an alternative to single-fraction stereotactic radiosurgery for the treatment of SBMs, especially for those close to the optic apparatus. With regard to the development of radiation-induced optic neuropathy, a similar risk <1% has been observed for maximum point doses to optic apparatus of 12 Gy given in one fraction, 20 Gy in three fractions, and 25 Gy in five fractions [113].

Over the last few decades, proton beam RT has been extensively employed in patients with skull base tumors with the rationale of better covering of the target while sparing surrounding critical structures compared to 3D-conformal RT and IMRT, especially in the case of large and irregularly shaped lesions [85]. Several studies of proton beam therapy for SBMs show 5-year local tumor control rates of 85–100% after either conventionally fractionated and hypofractionated schedules, being consistent with those observed following photon irradiation [101,102,103]. Using doses of 56 Gy, a variable occurrence of long-term side effects of 9 to 59% is reported in three studies including 136 patients (Table 3). In a small prospective study of 44 patients randomized to receive 55.8 Gy and 63.0 Gy (relative biological effectiveness, RBE) given as fractionated combined proton-photon RT, Sanford et al. [103] showed local control rates of 98% at 10 years and 90% at 15 years. With a median follow-up of 17 years, 26 patients (59%) experienced a grade 2 or higher late toxicity, including 9 patients (20%) who experienced a cerebrovascular accident. Currently, the superiority of proton beam therapy over advanced photon techniques in terms of efficacy and toxicity remains to be proven.

In summary, fractionated RT is a safe and effective technique for the treatment of patients with benign SBMs, with long-term local control consistent with those obtained following SRS. The choice of appropriate technique should be based on tumor size and site. In clinical practice, single doses of 8–10 Gy to the optic apparatus should be avoided to limit the risk of radiation-induced optic neuropathy. This means that SRS is usually suitable for patients with relatively small SBMs not in close proximity (less than 2 mm) to the optic apparatus, whereas fractionated schedules using either photons or protons would be preferred for tumors abutting the optic chiasm. Conventionally fractionated RT would be preferred over hypofractionated RT for larger tumors extensively involving the optic apparatus.

### 6.2. Radiosurgery

#### 6.2.1. Overview

SRS is nowadays widely accepted as a reliable alternative to microsurgery in selected cases [114], especially in the elderly and in tumors in critical locations, lowering mortality, morbidity and recurrences after surgery. The combined approach of subtotal resection and SRS post-operative treatment is of increased use.

Cranial nerve preservation is of utmost importance and in this clinical setting, SBMs represent the milestone of this phenomenon.

In the last decade, an increasing number of SBMs closer to critical brain structures such as the anterior optic pathways, brain stem, etc., have undergone SRS more and more often thanks to the introduction of innovative “volume staging” and “hypo-fractionated” irradiation techniques and modalities [110].

The excellent effectiveness and safety of SRS are thus reported, with a described 5-year actuarial progression-free survival (PFS) and local tumor control rates (LTR) of 86.2–97.9% [115,116,117] with very low sequelae [118,119,120], if appropriate indications are warranted, particularly regarding tumor volume and cytologic grading.

#### 6.2.2. Posterior Fossa

As is well-known, the posterior fossa presents some unique anatomical features resulting in a tiny space for mass effect. Surgical and radiosurgical features’ attitudes are therefore peculiar. In the literature, despite its enormous development, surgery results in mortality and morbidity (ranging in various studies from 40% to 96%), and could lead to recurrence after subtotal removal. Even if resection remains a class-A choice in cases with appreciable mass effect, a multimodality post-surgical approach is strongly recommended. Recent papers have even proposed conservative subtotal resection leading to the relief of mass effect and avoiding neurological injury, with SRS on the remnant [121]. In addition, posterior fossa meningiomas today are found at earlier stages of growth due to MRI availability and spreading. These are frequently asymptomatic or associated with minimal symptoms. Control rates reported following SRS have proved to guarantee a high tumoral control rate both in post-operative recurrences and in newly diagnosed, small non-symptomatic meningiomas [122,123,124] with progression-free survival rates greater than 90% [125,126]. Prognostic factors described in the literature for failures include age greater than 65 years, prior history of radiotherapy, and increasing tumor volume [126,127].

Moreover, predictors of neurological deterioration after radiosurgery could include large tumor volume, clival location or cerebellopontine angle (as opposed to tentorium or foramen magnum), but these last factors show a low statistical relation. On the contrary, tumoral shrinkage after 3 years from radiosurgery and a dose >16 Gy have been demonstrated to be positive prognostic factors [121].

#### 6.2.3. WHO Grade II and III

WHO Grade II meningiomas appear much less effective to SRS. The reported 5- and 10-year LTC rates are much lower (49–77% and 0–24%, respectively) [128,129]. In the past few years, these results caused fractionated RT to be advocated as an adjuvant or salvage treatment of these neoplasms [130].

If we consider the recent literature, however, SRS proved to be safe and effective for biopsy-proven WHO Grade II meningiomas. Adjuvant SRS following STR in small remnants or small surgical beds resulted in equivalent rates of long-term LTC as adjuvant RT.

Finally, higher radiation doses similar to those applied for malignant tumors should be recommended when possible for SRS treatment of atypical MNs [131].

#### 6.2.4. Combined MS–SRS Approach

A “combined Microsurgical-SRS approach” consists of a deliberate subtotal surgical resection, leaving a remnant near critical structures, followed by SRS [132]. A partial resection, due to its close-fitting position to neurovascular structures, results in increasing tumoral recurrence [133]. Therefore, in cases of atypical or anaplastic meningiomas, radiosurgery always has to be taken into account as an option after surgical debulking [134,135]. In cases of cavernous sinus or/and Meckel’s cave involvement in the skull base, a complete surgical resection might not be reliable, or very dangerous [12]. Common surgical strategies include resecting the maximal part of the tumor, followed by SRS on the remnant, especially if critically located. Radiosurgery in an early post-operative stage is nowadays routinely performed in patients with Grade 1 MN after incomplete resection [119,120,121,136,137]. A combined MS–SRS strategy has proved to be particularly worthy in cases of SBMs [137,138] and in some centers, this strategy is decided with the patients before surgery [138]. On the other hand, several studies have reported a progression of an untreated remnant for which a “wait and scan” policy is adopted [136].

Therefore, in cases of a surgical remnant, an early SRS might be planned in Grade 1–Grade 2 MNs, in order to avoid recurrence [139].

#### 6.2.5. Long-Term Follow-Up

In fact, many studies described excellent short to intermediate period results with 5- and 10-year LTR rates ranging from 86% to 100% and from 69% to 97%, respectively [140,141].

Kondziolka et al. [142] published a retrospective study on meningioma patients treated with GK (gamma-Kinife) SRS (70% of them located on the skull base and 97% WHO Grade I or with typical imaging features of a benign MN). The overall LTC rate was 91%. The 10- and 20-year actuarial rates of freedom from tumor progression of the targeted tumor after SRS was 85.3% ± 2.9% at both time points.

The long-term risk of severe permanent side effects following the SRS for SKMs is another controversial issue.

Recently, McClelland et al. [143] presented the results of an extensive analysis on the risk of stroke after SRS. On a total of 1431 patients followed up for a median/mean interval ranging from 75 to 144 months, 24 patients suffered a stroke following SRS, providing a stroke rate of 1.7%. This risk proved to be 12 times lower than the risk that occurred after fractionated proton-photon RT, and was comparable to that expected for the general population. Thus, SRS appeared to have the same stroke risk profile as observation.

Recently, Talacchi et al. published a robust retrospective analysis on 170 cavernous sinus meningiomas treated with GK SRS and followed up for at least 10 years. The LTC rate at 15 years after SRS was 89%. Neurological status was stable or improved in 147 patients (86.5%), independently of tumor shrinkage. WHO Grade I vs. Grade II histology (*p* = 0.019) was proven to be the only independent variable for LTR [137].

Overall, these studies with long-term periods of observation for SBMs treated with SRS led to the conclusion that long-term LTC rates were sustained at intervals of more than 10 years after SRS, as well [143].

## 7. The Role of Systemic Treatments

Systemic treatment in meningiomas should be considered for recurrent disease when a surgical or radiotherapy approach cannot be considered. There is little evidence in the literature to support a systemic therapeutic treatment, with few cases and with scarce prospective data. However, the greater knowledge of the molecular and genetic aspect of this type of tumor has led to the experimentation of new systemic therapeutic strategies. Hydroxyurea is the most studied drug in this setting: the results of some retrospective studies appear to be limited and with contradictory results [144,145,146]. An Italian randomized study analyzed the association of hydroxyurea with or without imatinib for recurrent or progressive meningiomas: despite the small number of patients enrolled, the arm with hydroxyurea alone showed a trend for more activity [147]. The possibility of adding a systemic treatment as adjuvant therapy after surgery was also evaluated in a small prospective study that enrolled 14 patients, treated with cyclophosphamide, adriamycin and vincristine after surgery and radiotherapy; this approach demonstrated moderate efficacy with partial response and disease stability in 3 and 11 patients, respectively, obtaining a median overall survival (OS) of 5.3 years (95% CI, 2.6–7.6) and a progression-free survival of 4.6 years (95% CI, 2.2–7.1) [148]. In relation to the high expression of progesterone receptors in meningioma, some hormonal agents have been studied as possible systemic therapy: discrete rates of disease control were obtained with tamoxifen in a phase II study in unresectable and refractory meningioma [149], while in a larger, randomized, phase III study (SWOG S9005) [150], mifepristone, an anti-progestogen agent, demonstrated no significant benefit in overall survival compared to placebo alone [151]. Another type of therapeutic approach evaluated in clinical studies is the use of somatostatin analogs; in a small study, these drugs demonstrated moderate activity in 10/16 treated patients [152], while in another prospective phase II study, no benefit in terms of activity was found other than a best response of disease stability [153]. A recent study evaluated the association of octreotide with everolimus, an mTOR inhibitor, in recurrent or relapsed meningioma, ineligible for further surgery/radiotherapy. Enrolled patients received octreotide (30 mg/d, day 1) and everolimus (10mg/d, days 1–28) and the primary endpoint of the study was 6mPFS; a total of 20 patients were enrolled, including 2 WHO grade I meningiomas, 10 with WHO grade II and 8 with WHO grade III meningiomas (4 patients harbored *NF2* germline mutation). The 6mPFS was 55% (95%CI 31.3–73.5%). In 78% of patients, a >50% decrease in tumor growth rate at 3 months was observed [154]. The high expression of pro-angiogenic factors in meningiomas has led to research on possible anti-angiogenic therapies: in a phase II study, the efficacy of sunitinib, a multi-kinase inhibitor of VEGFR and PDGFR, was evaluated in patients with grade II and grade III meningiomas, reporting a median PFS of 5.2 months (95%CI 2.8–8.3), median OS of 24.6 months (95%CI 16.5–38.4) and the 6m-PFS rate was 42% [155]. In another retrospective study, the role of bevacizumab, a monoclonal antibody directed against the vascular endothelial growth factor VEGF, was evaluated, demonstrating a 6m-PFS of 43.8% (95%CI, 15.7–69.1) in high-grade meningiomas [156]. In a further phase II study, bevacizumab was tested in 40 patients with WHO Grade I–III meningioma patients, showing a partial response (PR) in only 5% of atypical meningiomas as the best response to treatment and stable disease (SD) in 100%, 85% and 82%, respectively [157]. Combination therapies with bevacizumab were tested, particularly in a phase II study in which bevacizumab was combined with everolimus, an inhibitor of the mTOR pathway, in WHO Grade I–III progressive/refractory meningiomas; this study demonstrated that the drug combination resulted in stable disease (SD) as the best response in 15 of 17 enrolled patients (88%) with 6 patients having a stable disease duration >12 months. Median PFS was 22 months (95% CI 4.5–26.8) and was found to be superior in patients with grade II–III meningioma (22 months) compared to grade I tumors tumors (17.5 months) [158].

A recent comprehensive review of the literature ultimately concluded that, in view of the lack of clinical evidence on improved survival and related toxicity, the use of bevacizumab should be carefully and individually evaluated [159].

Other tyrosine kinase inhibitors have been evaluated as possible treatments in patients with progressive meningioma: Vatalanib, a multi-targeted tyrosine kinase inhibitor, was tested in a phase II study in patients with WHO grade II and III meningioma. This study demonstrated that 6m-PFS was found to be 64% and 37.5% in grade II and III meningiomas, respectively, with a median PFS of 6.5 for grade II meningiomas and 3.6 months for grade meningiomas, respectively. The median OS was 26 months and 23 months for grade II patients and for patients with grade III histology, respectively [160].

The better understanding of the molecular and genetic aspects of meningioma has led to the development of several trials on personalized therapy: there is an ongoing phase II trial (NCT02523014) with Vismodegib (inhibitor of Hedgehog signaling pathways used in the treatment of basal cell carcinoma) associated with a FAK focal adhesion kinase inhibitor (GSK2256098SMO) in mutated *SMO/PTCH1* meningiomas. Meningiomas are the second most common tumor in patients diagnosed with neurofibromatosis (NF) 2; in these cases, the loss of NF2 expression is associated with the activation of the mTOR pathway.

Virtusertib, a dual mTORC1/mTORC2 inhibitor, was evaluated in a phase II trial in 18 patients; the treatment was associated with an objective response rate in 5–10% in NF2 meningioma and schwannoma [161]. Noteworthily, Trabectedin, an alkaloid antineoplastic drug approved for treatment of soft tissue sarcoma and ovarian cancer [162,163], was tested in the Phase II EORTC 1320 trial in adult patients with WHO grade II or III meningioma, after surgery and radiotherapy, and did not improve PFS and OS with greater toxicity than local standard of care [164].

Due to excellent results obtained with immunotherapy in some types of tumors, several ongoing trials are also testing the efficacy of nivolumab (NCT02648997) and pembrolizumab (NCT03016091; NCT03279692) in patients with recurrent high-grade meningioma.

## 8. Current Thinking and Future Perspectives

In most cases, intracranial meningiomas can be considered benign lesions. However, 10-year tumor recurrence rate ranges from 10 to 32% and surgical mortality rate can account up to 14.3% [79].

In summary, SBM treatment relies upon a multidisciplinary approach. First-line treatments include surgery for symptomatic SBMs, followed by (in selected cases) fSRT or SRS.

The postoperative treatment is individualized according to a combination of different clinical, radiological, surgical and histological factors.

**Figure 3:**
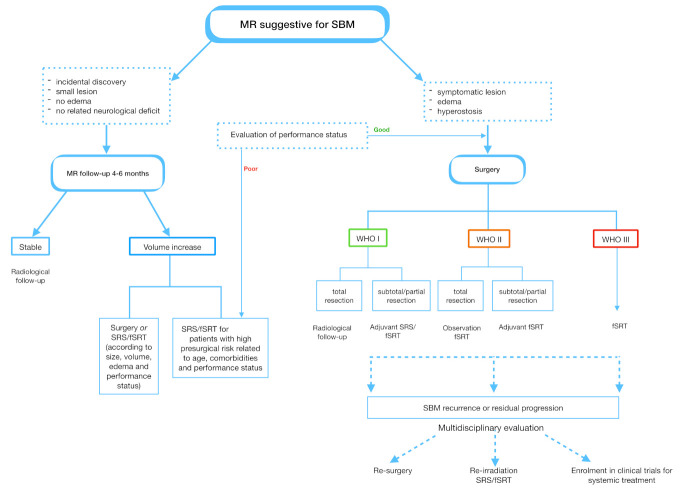
Workflow for the standard treatment options in SBMs. When a “wait and see” approach is chosen, MRI is performed every 6 months. If patients remain asymptomatic, MRI is performed annually after 5 years. For WHO grade III SBMs, fSRT is recommended across all surgical resection classes. WHO, World Health Organization; fSRT, fractionated stereotactic radiotherapy; SRS, stereotactic radiosurgery.

Figure 3 provides an overview of current advances in multidisciplinary management of SBMs.

The current literature shows that SRS and fSRT constitute a safe and efficient treatment option in SBMs both as an alternative to surgery in selected cases, and as adjuvant post-operative complementary treatment in accordance with the histological grade and the extent of resection achieved. Specifically, Grade II meningiomas represent a highly heterogeneous group with different clinical behavior that still lack therapeutic postoperative guidelines [89,90,91].

To date, pharmacological alternatives, including immunotherapy, have been developed but none of them have demonstrated a significant outcome benefit. Future clinical trials, designed in accordance with molecular characterization, could define a novel treatment classification. Molecular characterization of meningiomas based on NF2, but also *AKT, SMO, TRAF7* status, detects the subgroup of meningiomas with different clinical behavior, posing thus the bases for a novel meningioma classification. Recently, methylation profiles of meningiomas seem to very accurately predict tumor behavior; for example, the recurrence risk in olfactory groove meningiomas shows a significant correlation with SMO mutations. The latter mutations could be targeted by specific molecules [165,166].

In terms of RT, the future trials should define the proper dose-scheduling (higher or lower dose for primary or adjuvant approach) based on molecular behavior. Moreover, the use of proton therapy should be integrated in SBM management (aggressive histology, progressive or recurrent lesions) especially for the lower dose delivered to the surrounding healthy brain tissue (i.e., optic nerve and the brainstem) [165].

Although still in the beginning stages, these complementary molecular perspectives integrated into the classification could be associated with innovative and personalized treatment approaches [14,166].

## 9. Conclusions

Understanding the pathological anatomy and performing a preoperative anatomo-radiological evaluation is of utmost importance in the surgical planning and management of SBM.

Considering the technical challenges in SBM surgery, a multimodal treatment, in combination with radiosurgery and radiation therapy, is considered more and more to achieve a satisfactory functional outcome and tumor control. Advances in technology, genomics and radiomics could lead to enhanced profiling of tumor biology, with consequent refinement of treatment according to the principles of precision medicine.

In light of all these innovations, a specialized multidisciplinary approach is mandatory in SBM management, paving the way for “Centers of excellence”, ensuring an adequate workload and appropriate technological resources.

## Figures and Tables

**Table 1:** Literature review of surgical approaches according to SBMs location. Surgical approach is selected by interdisciplinary consultation according to the site of disease.

Skull Base Region	Location	Incidence	Surgical Approaches	IONM	Surgical Pitfalls	Complications		
					Vascular	Nerves	Others	Clinical Manifestations
Anterior Fossa 4, 27–36,49,51	Olfactory Groove Meningiomas	8–13%	Subfrontal approach Transbasal Approach Pterional approach Fronto-lateral approach Supraorbital keyhole Endoscopic endonasal approach *Transfacial reconstitutive approach also known as facial translocation, further subdivided into: - nasal cheek flap - maxillary cheek flap - nasomaxillary cheek flap - facial split (resulting from the combination of the aforementioned)	EEG, MEPs, SSEPs, VEPs (in selected cases)	branches of the OA, ICA, ACoA, A2 Ethmoidal arteries	CN I, II, III, IV	EOM	Anosmia, CSF leak Visual disturbances (diplopia, anopsia, eye globe injury) Hemorrhage Hemorrhage, epiphora, diplopia and dystopia, soft tissue swelling, ectropion (associated with Weber–Ferguson incision). Poor bone consolidation, misalignment (related to bad osteosynthesis), wound dehiscence (cutaneous and intraoral)
	Sphenoid wing Meningiomas	11–20%	Pterional approach Fronto-temporal-orbito-zygomatic approach Lateral, superior, medial orbitotomy		anterior circulation arteries Ethmoidal artery in medial accesses	CN II, III, VI supraorbital nerve, facial nerve	EOM, medial and lateral canthal tendons	
	Tuberculum Sellae/Planum Meningiomas	5–10%	Pterional approach Endoscopic endonasal approach Supraorbital key-hole		Anterior circulation arteries	CN II, III, IV, V, VI	aesthetic orbital reconstruction	
	Cavernous Sinus Meningiomas	1%	Pterional approach Fronto-temporo-orbito- Zygomatic approach		Anterior circulation arteries	CN II, III, IV, V, VI		
Middle Fossa 4,35,44	Middle fossa and Sphenoid wing	1.1–1.4%	Pterional approach (anterolateral approach) Fronto-temporo-orbito- zygomatic	EEG, MEPs, SSEPs EMG CNs III, IV, VI can be considered	ICA Vein of Labbè	CN II, III, IV, V, VI	Temporal lobe	Language deficit, hemiparesis, hemianopsia, hemorrhages, temporal lobe edema, trigeminal anesthesia,
	Middle fossa and cavernous sinus							
	Middle fossa with infratemporal extension		Subtemporal approach (lateral approach)					
	Middle fossa and petrous ridge							
Posterior Fossa 4,35,38–43,45,46,51	Cerebellopontine Angle	10%	Anterior Petrosectomy Approach Posterior and Combined Petrosal Approaches Retrolabyrinthine Approach Translabyrinthine Approach Combined Petrosal Approach Retrosigmoid approach	EEG, MEPs, SSEPs, CB-MEP (CN VII) EMG (CNs VI, VII) BAERs	Intrapetrous ICA, SCA and AICA encasement	CN V, VI, VII, VIII	Brainstem adhesion	Brain steam and cerebellum edema, CSF leak Venous infarction Cranial nerve injury Vertebral artery injury, Hydrocephalus, CSF leak Infection Hemorrhage, cerebrospinal fluid leakage, soft tissue edema of the oral cavity, infection, wound dehiscence, velopalatine dysfunction, malocclusion, dysphagia, malocclusion when osteotomies are required, oro-nasal fistula, laceration of nasal mucosa, lesion of teeth apices
	Foramen Magnum	2.5%	Posterior Suboccipital Approach with C1 laminectomy; Far Lateral Approach Extreme Lateral Approach	EEG, MEPs, SSEPs, CB-MEP (CN VII, IX, X, XI, XII) EMG (CNs VI, VII, IX, X, XI, XII) BAERs	VA encasement JV encasement	CN IX, X, XI, XII	Brainstem adhesion Extradural extension	
	Clival Meningiomas	<1%	Retrosigmoid approach Petrosal approach Transoral: - transmaxillary through LeFort I osteotomy - transmaxillary with palatal split posterolateral approach far-lateral approach Endoscopic approach		Internal maxillary artery Palatine artery	CN VI, VII, VIII, XI, X, XI, XII	Brainstem adhesion	
	Petroclival Meningiomas	2% of posterior fossa meningiomas	Retrosigmoid approach Combined transpetrosal Retrolabyrinthine Approach Translabyrinthine Approach		BA BA perforating arteries	CN V, VI, VII, VIII	Brainstem adhesion	

Legend: * alone or in combination with bifrontal craniotomy for selected wide Olfactory Groove meningiomas invading cribriform plate; OA: ophthalmic artery; ICA: internal carotid artery; ACoA: anterior communicating artery; A2: second segment of anterior cerebral artery; ACA: anterior cerebral artery; SCA: superior cerebellar artery, AICA: anterior inferior cerebellar artery, VA: vertebral artery; BA: basilar artery; JV: jugular vein; CN: cranial nerve; EOM: extraocular muscles; IONM: intraoperative nerve monitoring; BAERs: brain stem auditory-evoked responses; CB-MEPs: corticobulbar motor-evoked potentials; CNs: cranial nerves; EEG: electroencephalogram; EMG: electromyography; MEPs: motor-evoked potentials; SSEPs: somatosensory-evoked potentials; VEPs: visual-evoked potentials.

**Table 2:** Genetic alterations in skull based meningiomas.

Altered Gene	Preferential Tumor Localization	Main Histotype
*NF2*	Posterior and lateral skull base	Fibrous, Transitional, Atypical
*AKT1, PI3K*	Anterior and middle skull base	Meningothelial
*SMO*	Olfactory groove	Meningothelial *
*TRAF7/KLF4*	Middle skull base	Meningothelial, Secretory for co-occurring *TRAF7/KLF4*
*POL2RA*	Tuberculum sellae	Meningothelial

* *SMO* mutated meningiomas have significantly higher recurrence risk than *AKT1* meningiomas at the same site.

**Table 3:** Summary of selected published studies on conventionally fractionated radiotherapy for benign SBMs.

Authors	Patients (N)	Radiation Modality	Median Dose/ Dose per Fraction (Gy)	Median Volume (mL)	Median Follow-Up (Months)	Local Control	Late Toxicity (%)
Goldsmith et al., 1994 [79]	117	CRT	54	NA	40	89 at 5 and 77 at 10 years	3.6
Maire et al., 1995 [80]	91	CRT	52	NA	40	94	6.5
Nutting et al., 1999 [81]	82	CRT	55–60	NA	41	92 at 5 and 83 at 10 years	14
Vendrely et al., 1999 [82]	156	3D-RT	50	NA	40	79 at 5 years	11.5
Mendenhall et al., 2003 [83]	101	3D-RT	54	NA	64	95 at 5, 92 at 10 and 15 years	8
Henzel et al., 2006 [93]	84	fSRT	56	11,1	30	100	NA
Tanzler et al., 2010 [94]	144	fSRT	52.7	NA	87	97 at 5 and 95 at 10 years	7
Minniti et al., 2011 [95]	52	fSRT	50	35.4	42	93 at 5 years	5.5
Slater et al., 2012 [101]	68	Protons	56	27.6	74	99 at 5 years	9
Weber et al., 2012 [102]	24	Protons	56/1.8–2.0	21.5	62	100 at 5 years	15.5
Solda et al., 2013 [96]	222	fSRT	50/55	12	43	100 at 5 and 10 years	4.5
Combs et al., 2013 [97]	507	fSRT/IMRT	57.6/1.8–2.0	53.4	107	95.5 at 5 and 88 at 10 years	1.8
Fokas et al., 2014 [98]	253 *	fSRT	55.8/1.8–2.0	16	50	92.9 at 5 and 87.5 at 10 years	12 (G2)
Han et al., 2014 [100]	143	fSRT	50.4/1.8	11.1	32	95%	0.7
Kaul et al., 2014 [104]	136	fSRT	57/1.8–2.0	24	44.9	93.8 at 5 and 91.5 at 10 years	G1 only
Sanford et al., 2017 [103]	44	Protons	55.8–63	39.7/13.2	195	98 at 10 and 90 at 15 years	59% (≥G2)
Lillie O’steen et al., 2019 [84]	149	3D-RT	50–52/1.7–1.8	NA	144	95 at 10 and 92 at 20 years	NA

CRT, conventional radiation therapy; fSRT, fractionated stereotactic radiation therapy; IMRT, intensity modulated radiation therapy; G, grade; 3D-RT, three-dimensional conformal radiation therapy; * series including skull base and intracranial meningiomas; NA, not assessed.

**Table 4:** Summary of selected published studies on conventionally fractionated radiotherapy for skull base meningiomas.

Authors	Patients (N)	Technique	Median Dose (Gy)/Fractions	Median Volume (mL)	Median Follow-Up (Months)	Local Control	Late Toxicity
Colombo et al., 2009 [108]	150 *	CK	16–25/2–5	7.5 (0.1–64)	30	96	3.5
Fokas et al., 2014 [98]	49 *	LINAC	25–35/5	6.11 (1.9–35.7)	50	92.9 at 5 and 87.5 at 10 years	12 (G2)
Han et al., 2014 [100]	22 *	LINAC	25/5	4.8 (0.88–20.38)	32	95	0.7
Navarria et al., 2015 [109]	26	LINAC	25/5	13°	24.5	93% at 2 years	G3, none
Marchetti et al., 2016 [110]	143	CK	21–25/2–5	8 (0.1–126.3)	44	93 at 5 years	5.1
Marchetti et al., 2019 [111]	168	CK	25/5	7.3 (0.1–76.8)	51	94% at 5 years	3.7

CK, CyberKnife; LINAC, linear accelerator; mean; * including skull base and intracranial meningiomas.
